# Hsp90 and Associated Co-Chaperones of the Malaria Parasite

**DOI:** 10.3390/biom12081018

**Published:** 2022-07-22

**Authors:** Tanima Dutta, Harpreet Singh, Adrienne L Edkins, Gregory L Blatch

**Affiliations:** 1 The Vice Chancellery, The University of Notre Dame Australia, Fremantle, WA 6160, Australia; tanimadutta85@gmail.com; 2 The Institute of Immunology and Infectious Diseases, Murdoch University, Perth, WA 6150, Australia; 3 PathWest Nedlands, QEII Medical Centre, Perth, WA 6009, Australia; 4 Department of Bioinformatics, Hans Raj Mahila Maha Vidyalaya, Jalandhar 144008, India; harpreetsingh05@gmail.com; 5 Biomedical Biotechnology Research Unit, Department of Biochemistry and Microbiology, Rhodes University, Grahamstown 6140, South Africa; a.edkins@ru.ac.za; 6 Biomedical Research and Drug Discovery Research Group, Faculty of Health Sciences, Higher Colleges of Technology, Sharjah P.O. Box 7947, United Arab Emirates

**Keywords:** *Plasmodium falciparum*, heat shock proteins, cytosolic Hsp90, ATPase, co-chaperones, client proteins

## Abstract

Heat shock protein 90 (Hsp90) is one of the major guardians of cellular protein homeostasis, through its specialized molecular chaperone properties. While Hsp90 has been extensively studied in many prokaryotic and higher eukaryotic model organisms, its structural, functional, and biological properties in parasitic protozoans are less well defined. Hsp90 collaborates with a wide range of co-chaperones that fine-tune its protein folding pathway. Co-chaperones play many roles in the regulation of Hsp90, including selective targeting of client proteins, and the modulation of its ATPase activity, conformational changes, and post-translational modifications. *Plasmodium falciparum* is responsible for the most lethal form of human malaria. The survival of the malaria parasite inside the host and the vector depends on the action of molecular chaperones. The major cytosolic *P. falciparum* Hsp90 (PfHsp90) is known to play an essential role in the development of the parasite, particularly during the intra-erythrocytic stage in the human host. Although PfHsp90 shares significant sequence and structural similarity with human Hsp90, it has several major structural and functional differences. Furthermore, its co-chaperone network appears to be substantially different to that of the human host, with the potential absence of a key homolog. Indeed, PfHsp90 and its interface with co-chaperones represent potential drug targets for antimalarial drug discovery. In this review, we critically summarize the current understanding of the properties of Hsp90, and the associated co-chaperones of the malaria parasite.

## 1. Introduction

To combat cellular stress, an elevated expression of chaperones, many of which are heat shock proteins, is observed [1]. In eukaryotes, heat shock protein 90 (Hsp90) and heat shock protein 70 (Hsp70) are the most prominent chaperone families. Together, Hsp90 and Hsp70 collaborate to ensure protein homeostasis by capturing client proteins and facilitating productive folding [2]. Hsp90 has essential functions in cell growth and differentiation, apoptosis, signal transduction, and cell–cell communication [3]. Hsp90 isoforms exist in organisms ranging from bacteria (where it is known as HtpG) to protozoa to higher eukaryotes. Although Hsp90 is not essential for cell survival in the bacterium *Escherichia coli*, it is important for the survival of *Shewanella oneidensis* under heat stress [4]. It is indispensable for viability in the yeast *Saccharomyces cerevisiae* [5], while in higher eukaryotes the Hsp90β, but not the Hsp90α, isoform is essential for survival [6,7,8,9]. Hsp90 plays a central role in many cellular networks, along with buffering environmental conditions to promote evolutionary fitness [10].

*Plasmodium falciparum* is responsible for the most lethal form of human malaria, taking 627,000 lives worldwide in 2020 [11]. Infection begins with a female mosquito injecting sporozoites into human blood. Following the mosquito’s ‘blood meal’, the successful colonization of the liver by sporozoites initiates the parasite life cycle in humans, followed by erythrocyte invasion, which accounts for the pathology of malaria [12,13]. The development of sporozoites takes place within hepatocytes, where they mature into schizonts and then merozoites, which are released and rapidly invade erythrocytes [13,14]. The intra-erythrocytic stage results in alterations of the infected host cells that cause them to adhere to the cell walls of capillaries, thereby preventing them from clearing through the spleen. This structural change poses a risk for the human host, since clusters of infected erythrocytes can create a blockage in blood circulation. After the intra-erythrocytic stage, the gametocyte-infected stage develops, which can infect the mosquito upon blood ingestion [12]. The motile ookinetes penetrates the midgut wall of the mosquito, developing into “oocysts”. These cysts then release sporozoites, which migrate to the mosquito’s salivary glands and can again infect the human host [12]. During the intra-erythrocytic stage, high temperatures are induced and, therefore, parasite proteins and membranes require cytoprotection for the maintenance of their integrity [15]. Survival of the malaria parasite inside the host and the vector depends on the action of molecular chaperones. The emergence of resistance to the most commonly used antimalarial drugs, coupled with the difficulty in producing an effective vaccine, resulted in an urgent need to develop drugs targeted against novel chemotherapeutic targets [16,17,18].

There is evidence from saturation-scale mutagenesis screening that all the Hsp90 genes of the malaria parasite are essential [19]. Furthermore, the major cytosolic *P. falciparum* Hsp90 (PfHsp90) is highly expressed during the intra-erythrocytic stage of the parasite life cycle, induced by stress, and plays an essential role in parasite survival and development [7]. Using in vitro cell culture studies, geldanamycin (GA) was found to be highly effective at inhibiting the growth of parasite-infected erythrocytes, and causing an arrest at the ring stage [7,20]. Assuming that PfHsp90 was the primary target of GA, these findings suggest that PfHsp90 plays an important role in malaria parasite growth in erythrocytes [7,20]. In addition, given that transition from early ring to metabolically active trophozoites is regulated by temperature changes, PfHsp90 was also proposed as a major player in the malaria parasite’s response to heat shock, and the establishment of infection in erythrocytes [21,22]. Indeed, frequent febrile episodes elevate the level of PfHsp90 expression, and GA inhibition studies suggest that PfHsp90 assists in malaria parasite survival during febrile episodes [23,24]. Interestingly, PfHsp90 is also shown to be essential for liver stage development [25]. Overall, these findings suggest that PfHsp90 is an ideal anti-malaria drug target.

## 2. Hsp90: Chaperone Activity and Its Conformational Changes

**Figure 1:**
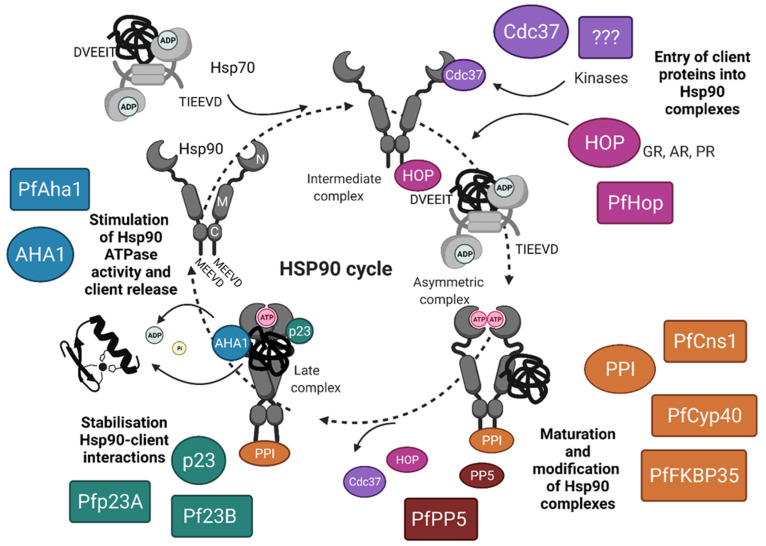
Regulation of the Hsp90 chaperone cycle by co-chaperones. Progression of client proteins through the Hsp90-mediated chaperone folding pathway is regulated by co-chaperones, which act at defined stages in the cycle. Co-chaperones may regulate Hsp90 association with clients, ATPase activity, conformational changes, and post-translational modifications. When inactive, Hsp90 is constitutively dimerized at the C-terminus but not the N-terminus. Entry of client proteins is facilitated by co-chaperones including the Hsp70/Hsp90 organizing protein Hop, which regulates transfer of clients from Hsp70 by binding simultaneously to Hsp70 and Hsp90, to form the intermediate complexes. Hop is conserved in *Plasmodium falciparum* (PfHop, PF3D7_1434300). Kinase clients require the kinase-specific co-chaperone Cdc37; however, a Cdc37-encoding gene has not been identified in the *P. falciparum* genome. On ATP binding, Hsp90 undergoes N-terminal dimerization, and the client protein associates with the middle domain of Hsp90. Bindings of other co-chaperones, including peptidyl-prolyl cis–trans isomerases (PPIase) and protein phosphatase 5 (PP5), associate to form the asymmetric Hsp90 complexes. The *P. falciparum* genome encodes a PP5 isoform (PfPP5, PF3D7_1355500) and multiple PPIase isoforms (PfFKBP35, PF3D7_1247400; PfCns1, PF3D7_1108900; and PfCyp40, PF3D7_1111800). These co-chaperones regulate the post-translational modification and maturation of Hsp90 complexes. Early co-chaperones subsequently dissociate from the complex to be replaced by p23, which stabilizes the late closed Hsp90 complex and the client within the complex, and inhibits ATPase activity. Two homologs of p23 are encoded in the *P. falciparum* genome (Pfp23A, PF3D7_1453700; and Pf23B, PF3D7_0927000). ATP hydrolysis is stimulated by binding of Aha1, resulting in release of the client protein and a return of Hsp90 to the inactive conformation. The *P. falciparum* genome encodes a single Aha1 isoform (PfAha1, PF3D7_0306200). Image created with BioRender.com.

**Figure 2:**
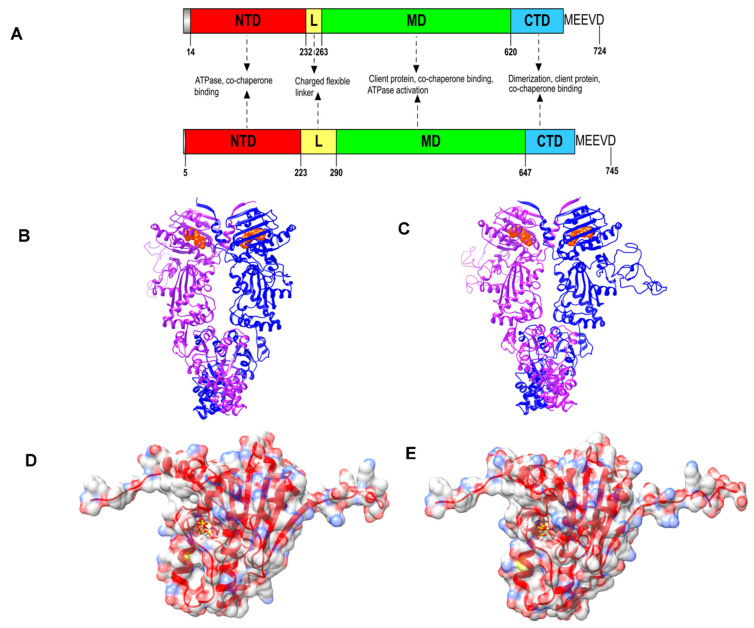
Domain organization and structural view of hHsp90β and PfHsp90. (**A**). Domain organization of hHsp90β (**top**) and PfHsp90 (**bottom**). Structure of full-length dimeric (**B**). hHsp90β and (**C**). PfHsp90 proteins as cartoons. ATP bound to the N-terminal domain (NTD) is shown as red spheres. The two Hsp90 monomers in the models are colored purple and blue. (**D**). hHsp90β and (**E**). PfHsp90 NTD as surface. The surface (with 60% transparency) is colored according to element type and it also depicts the arrangement of secondary structure elements (red color) as cartoons. The bound ATP molecule is represented as sticks, colored according to the element type. Full-length 3D structures of hHsp90β and PfHsp90 were modeled with SWISS-MODEL (SWISS-MODEL: homology modelling of protein structures and complexes. Available online: https://swissmodel.expasy.org/ [accessed on 12 June 2022]) using PDB files 5FWK and 5FWM, respectively, as templates. NTD: N-terminal domain; L: linker region; MD: middle domain; and CTD: C-terminal domain. Element coloring scheme uses red, blue, grey, and yellow for oxygen, nitrogen, carbon, and phosphorous, respectively. Images for 3D structures were rendered using UCSF Chimera 1.10.1 (UCSF Chimera—a visualization system for exploratory research and analysis. Available online: https://www.cgl.ucsf.edu/chimera/ [accessed on 12 June 2022]), while the linear domain layout image was rendered using IBS 1.0 (IBS: an illustrator for the presentation and visualization of biological sequences. Available online: http://ibs.biocuckoo.org/ [accessed on 12 June 2022]).

Cytosolic Hsp90 architecture is conserved from bacteria to humans with slight modifications, which are critical for functional differences between Hsp90 paralogs and orthologs [26]. The most common structural feature of all Hsp90 homologs is the presence of an N-terminal nucleotide-binding domain (NTD), along with a C-terminal domain (CTD) and a middle domain (MD) [27] (Figure 1 and Figure 2; Protein Data Bank [PDB] identification [ID] codes: 5FWK and 5FWM). Hsp90 functions as a molecular machine to capture and promote the folding of client proteins through conformational changes regulated by ATPase activity and protein–protein interactions [28]. ATP binds the Hsp90 NTD, and ATP hydrolysis is catalyzed by the NTD and MD. The NTD and MD are joined by a charged linker sequence, which is important for inter-domain communication during chaperone activity [29].

The MD also carries the binding site for Hsp90 clients and co-chaperones. The CTD allows the constitutive dimerization of Hsp90 through two C-terminal helices forming a four-helix bundle [30,31]. One of the most prominent features of Hsp90 chaperone activity is the formation of a V-shape dimer, which helps in the transient N-terminal dimerization that is required for ATP hydrolysis [32] (Figure 1). A C-terminal MEEVD motif is present in all cytosolic Hsp90 paralogs, and is the main site of binding to tetratricopeptide repeat (TPR)-containing co-chaperones [33]. Co-chaperones of eukaryotic Hsp90s typically out-number their respective chaperones, forming complexes with Hsp90 and their client proteins, to promote efficient protein folding and fine-tuning chaperone functions to maintain cellular homeostasis (Figure 1). Consequently, new approaches to inhibit the function of the Hsp90 complex have focused on the disruption of protein–protein interactions with co-chaperones [34].

Hsp90 modulates the stability of several essential cellular proteins, and is a conserved regulator of key protein kinases and nuclear receptors that control the cell cycle and signal transduction events [35,36,37]. The NTD is rich in β-strands and forms a nucleotide-binding pocket sharing a Bergerat fold with members of the GHKL superfamily (gyrase subunit B [GyrB], histidine kinase, and DNA mismatch repair protein MutL) [38]. This domain can be inhibited competitively by small molecule inhibitors, which target the ATP binding site and, as such, compete with ATP for binding [38,39,40]. The NTD and MD of Hsp90 undergo key conformational changes, bringing the γ-phosphate of ATP closer to key residues in the MD (e.g., Arg380 in yeast Hsp82), which triggers ATP hydrolysis [41]. Also, Hsp90 has a much higher affinity for ADP than ATP, suggesting that it requires a threshold cellular ATP:ADP ratio for ATPase activity [39,42,43]. In general, all Hsp90s bound to ATP can associate with unfolded/partially folded client proteins. Subsequently, the lid region closes over the ATP binding pocket, and the NTD dimerizes, adopting a closed conformation. The association of the MD in the Hsp90 dimer alters the position of the MD catalytic loop promoting ATP hydrolysis (Figure 1). Upon ATP hydrolysis, the client protein is released to fold spontaneously [2]. The Hsp90 homodimer returns to the unbound open conformation, and is primed for subsequent rounds of ATP hydrolysis and protein folding [38].

## 3. *P. falciparum* Hsp90s

The *P. falciparum* genome contains four Hsp90 genes, encoding the following PfHsp90 proteins: PfHsp90 (cytosol; PF3D7_0708400), PfTrap1/PfHsp90_M (mitochondrion; PF3D7_1118200), PfGrp94 (endoplasmic reticulum; PF3D7_1222300), and PfHsp90_A (apicoplast; PF3D7_1443900) [44]. Low resolution structural studies suggest that PfHsp90 exists in solution as elongated and flexible dimers [37] (Figure 2). While PfHsp90 shares significant sequence and structural similarity with its eukaryotic homologs, particularly cytosolic human Hsp90β (hHsp90), and contains all the characteristic domains (NTD, charged linker region, MD, CTD, and C-terminal dimerization domain ending in a MEEVD motif), it has several key structural and functional differences [45,46,47] (Figure 2). In particular, the ATP-binding pocket of PfHsp90 is more hydrophobic, constricted, and basic, relative to hHsp90 [48]. Biochemical studies on PfHsp90 report that, in comparison to hHsp90, it binds ATP with higher affinity (by 30%), is a more active ATPase (with six-fold higher activity), and has significantly higher catalytic efficiency (*k*_cat_/*K_m_* of 16.2 × 10^−5^ min^−1^ µM^−1^) [49]. While basal ATPase kinetics and, ultimately, the speed of the chaperone cycle are important factors, they are not sufficient for efficient client protein folding by Hsp90 [50,51]. There is evidence that the dwelling time between the open and closed conformations of Hsp90 is critical to ensuring appropriate client protein interaction [50] (Figure 1); and, hence, more detailed biophysical studies are required on PfHsp90. Interestingly, PfHsp90 has a highly (negatively) charged, flexible linker region that is substantially longer than that of hHsp90 [52]. Domain swapping experiments introducing the charged linker from PfHsp90 into yeast or human Hsp90 lead to chimeric proteins, which support viability in yeast but have reduced ATPase activity, and reduced interaction with client proteins and some co-chaperones [52]. It remains to be determined how the intrinsic biochemical properties of PfHsp90 are regulated by different client proteins and their associated co-chaperones. Nevertheless, these initial biochemical findings suggest that the PfHsp90 chaperone cycle may be capable of rapid client protein turnover, which would be highly advantageous to parasite survival under the stressful conditions experienced in the human host. Furthermore, these unique architectural and biochemical features of PfHsp90 suggest that it is a prime drug target for structure-based anti-malarial drug discovery [53].

While co-chaperones of hHsp90 are extensively studied [53], and informed anti-cancer drug discovery [34], there are relatively few studies on PfHsp90 co-chaperones. Increasing our understanding of how PfHsp90 and its co-chaperones interact would greatly assist the development of novel anti-malarial therapies. Table 1 provides a comparison of the known co-chaperones of PfHsp90 to those of hHsp90, and in the following sections these proteins are explored in further detail.

## 4. PfHop (Hsp70–Hsp90 Organizing Protein; PF3D7_1434300)

As in other eukaryotes, the PfHsp70 and PfHsp90 protein folding pathways intersect to facilitate the folding of key proteins involved in diverse cellular pathways [22,46]. The interaction between Hsp70 and Hsp90 is regulated by Hop, which has been extensively characterized in the human system [68]. Both Hsp70 and Hsp90 possess C-terminally located EEVD motifs that interact with Hop via its multiple TPR domains [33]. Hop is not required for chaperone-mediated protein folding by Hsp70 and Hsp90 [69], but rather plays an important regulatory role for progression of client proteins through the chaperone cycle [70] (Figure 1). Of the six Hsp70-like proteins encoded by the *P. falciparum* genome, only the cytosol-nuclear localized chaperone PfHsp70-1 possesses the EEVD motif [71], which is crucial for interaction between Hsp70 and Hop. A Hop homologue (PF14_0324) was identified in the *P. falciparum* genome by Acharya and co-workers [44] (Table 1). Overall structural conservation was reported in PfHop, with some variations in the TPR regions [54]. Less conserved segments of Hop outside its TPR domains are shown to influence the overall conformation of the helical turns of the TPR domains, therefore, imparting unique structural features to Hop molecules from different species [72]. Immunofluorescence studies show PfHop to be localized with PfHsp70 and PfHsp90 in the parasite, and PfHsp70-1 complexes contained both PfHsp90 and PfHop by co-immunoprecipitation analysis [54]. PfHop co-localizes with the cytosolic chaperones PfHsp70-1 and PfHsp90 at the blood stages of the malaria parasite, and PfHop is stress-inducible [73,74]. Employing far western, surface plasmon resonance (SPR) and co-immunoprecipitation studies, a direct interaction between PfHop and PfHsp70-1 was identified, which was favored in the presence of ADP rather than ATP [73]. Recent studies on PfHop employing synchrotron radiation circular dichroism (SRCD) and small-angle X-ray scattering reveal that PfHop is a monomeric and elongated protein [55]. PfHop is also found to be unstable at temperatures higher than 40 °C in comparison to its functional partner, PfHsp70-1, which is known to be stable at temperatures as high as 80 °C [55,75].

## 5. PfTah1 (TPR-Containing Protein Associated with Hsp90; PF3D7_0213500) and PfPih1 (Protein Interacting with Hsp90; PF3D7_1235000)

The R2TP complex is an important multiprotein complex involved in multiple cellular process such as snoRNP biogenesis, PIKK signaling, RNA polymerase II assembly, and apoptosis [56]. Within the R2TP complex, the specialized Pih1 co-chaperone tightly interacts with Rvb1/Rvb2 and with another specialized co-chaperone Tah1 to form the R2TP macromolecular complex. The R2TP complex further interacts with Hsp90 to form the R2TP–Hsp90 complex [56]. A genome-wide screening of *P. falciparum* led to the identification of PfPih1 and PfTah1, which associate with PfHsp90 to form the *Plasmodium* R2TP–Hsp90 complex [47,56] (Table 1). The R2TP complex plays a vital role in both cancer cell proliferation in humans and rapid multiplication of *P. falciparum* [56].

## 6. Immunophilins: PfCyp40 (Cyclophilin 40/PF3D7_1111800) and PfFKBP35 (FK506-Binding Protein 35/PF3D7_1247400)

Immunophilins are known for their characteristic peptidyl-prolyl cis–trans isomerase (PPI) activity [76]. Cyclophilin 40 (Cyp40) and FK506-binding proteins (FKBPs) were discovered in 1989 as the major receptors of the immunosuppressive drugs Cyclosporine-A and FK506 (tacrolimus), respectively [77,78]. PPIs play an accessory role with the Hsp90 protein folding machinery, and are part of diverse intracellular signaling pathways, ranging from steroid receptors to regulatory tyrosine kinases, critical in cell cycle control [79,80]. In humans, Cyp40, along with FK506-binding proteins FKBP51 and FKBP52, are also components of steroid receptor complexes [81,82,83]. All three immunophilins (Cyp40, FKBP51, and FKBP52) have conserved N-termini for immunophilin function and a C-terminal domain containing TPR motifs involved in protein–protein interaction [83,84]. They all target Hsp90 through their conserved C-terminal region to form separate steroid receptor complexes containing Hsp90 (Figure 1). Smith and co-workers (1990) [85] explained the dynamic model of steroid receptor assembly, in which the high affinity hormone-binding form of the receptor was regulated through interactions between Hsc70 and Hsp90. The immunophilins are known to regulate the activity of steroid hormone receptors, and their interaction depends on the type of steroid hormone receptor to be activated. FKBP51 preferentially interacts with progesterone and glucocorticoid receptor complexes, while Cyp40 tends to accumulate with estrogen receptor complexes [86]. Mining of the *P. falciparum* 3D7 genome reveals eight putative cyclophilin chaperones with four α-like and four β-like subunits [87]. No *Plasmodium* export element (PEXEL) motifs were found in any of the putative cyclophilins co-chaperones. It was observed that only two have PPIase activity, but all of them prevent aggregation of a model substrate, and are implicated in heat shock resistance in *P. falciparum* [88]. *P. falciparum* Cyp40 (PfCyp40; Table 1) has a predicted C-terminal trans-membrane domain and no export signal [81]. Most of the PfCyps are identified as having no signal peptide and, therefore, would most likely be found in the parasite cytoplasm [89]. Similar to the mammalian counterpart, two PPIase monomers of PfCyp40 are predicted to interact with dimeric PfHsp90 [90].

One of the most highly expressed co-chaperones of hHsp90 across a range of tissues is FKBP38, a membrane-anchored protein distributed predominantly in mitochondria [91,92]. *P. falciparum* FKBP35 (PfFKBP35; Table 1), a putative FKBP38 homologue, is shown to be functional in that it exhibits PPIase activity that is sensitive to inhibition by FK506 and Rap [93]. Pull-down assays reveal that PfFKBP35 interacts with PfHsp90 through its TPR domain, suggesting that PfFKBP35 is a co-chaperone of PfHsp90 [94]. There is limited information on the exact mechanism of inhibitors such as FK506 in the interaction between PfFKBP35 and PfHsp90. PfFKBP35 itself might be responsible for the antimalarial effects of FK506 and Rap. Pharmaco-dynamics analysis suggests that both FK506 and Rap have similar effects on different intra-erythrocytic stages in culture and kinetics of killing or irreversible growth arrest of parasites [95]. Furthermore, X-ray and NMR crystallography experiments show slight differences between PfFKBP35 and another human PPI, FKBP12, which could be critical in the designing of inhibitors that selectively inhibit PfFKBP35 [96]. The structural differences were detected in the β5–β6 segment of the PPIase domain, where PfFKBP35 contains a conserved cysteine and serine residue at amino acid positions 106 and 109, respectively, instead of a histidine (H87) and isoleucine (I90) residue at the corresponding position in human FKBP12, which presents as an architectural FKBP domain. Another study on the design of small molecules, targeting these conserved C106/C105 and S109/S108 residues in PfFKBP35/*Plasmodium vivax* FKBP35 (PvFKBP35) to achieve selectivity, identified a novel ligand D44 (N-(2-Ethylphenyl)-2-(3H-imidazao [4, 5-b] pyridin-2-ylsulfanyl)-acetamide) with potent inhibitory activity against PfFKBP35 [97]. D44 displays approximately 100-fold higher selectivity towards the inhibition of *Plasmodium* FKBPs over human FKBPs (FKBP12 and FKBP52). Structural analysis reveals that the high selectivity towards *Plasmodium* FKBPs is attributed to improved proximity between D44 and the conserved C106/C105 and S109/S108 amino acid residues in PfFKBP35/PvFKBP35. In addition, another study proposed the incorporation of a bulky hydrophobic group at C-11 of FK506, to induce steric clashes with the residues H87 and I90 in FKBP12, as a potential strategy for engineering inhibitors that are selective towards PfFKBP35, while avoiding off-target effects on human FKBP12 [98].

## 7. Pfp23A (PF3D7_1453700) and Pfp23B (PF3D7_0927000)

The late stage co-chaperone p23 binds to the N-terminal domain of Hsp90, and is important for promoting the closed client-bound conformation of Hsp90 and inhibiting ATPase activity [70] (Figure 1). Pfp23, a 34-kDa phosphoprotein, is highly expressed and phosphorylated in the trophozoite stage of *P. falciparum* intra-erythrocytic development [99]. GST pull-down assays reveal the role of Pfp23 as a co-chaperone of PfHsp90, and this chaperone-co-chaperone interaction is dependent on the presence of ATP [61]. This is similar to the association between Sba1 (p23 yeast homologue) and yeast Hsp90 [100]. More recently, two small acidic co-chaperones, p23 orthologues, were identified in the *P. falciparum* genome [62] (Table 1). It was revealed that Pfp23A and Pfp23B show 13% identity between themselves, and 20% identity with human p23. It was found that Pfp23A has higher thermal stability in comparison to Pfp23B, suggesting structural and functional variability [62]. Both Pfp23A and Pfp23B could inhibit PfHsp90 ATPase activity, although Pfp23A was more effective [62], and although both could prevent aggregation of model substrate proteins (malate dehydrogenase, citrate synthase, and luciferase), the isoforms showed preferences for model client proteins [62]. Site-directed mutagenesis experiments by Chua et al. [61] identified the conserved residues K91, H93, W94, and K96 in Pfp23 as critical for interaction with PfHsp90. Pfp23 was also found to suppress protein aggregation dependent on to its C-terminal tail, showing that it has chaperone activity independent of PfHsp90 [61]. In a separate study to screen cancer inhibitors, the anticancer compound gedunin was identified as a specific inhibitor of p23 [101]. Gedunin binds p23 and abrogates interaction with Hsp90, resulting in cancer cell death. Although gedunin was previously shown to inhibit the chaperone function of Hsp90, the precise inhibitory mechanism is unclear, as gedunin does not bind to the N-terminus or the C-terminus of Hsp90 as most Hsp90-specific inhibitors do (e.g., ansamycin antibiotics, radicicol, and novobiocin) [102,103]. In addition, gedunin shows antimalarial activity, which may or may not be related to its ability to modulate the interaction of Pfp23 and Hsp90 [104]. The presence of two Pfp23 isoforms with putative functional differences is interesting, and suggests that the mechanism of stabilization of PfHsp90 late stage complexes differs from that of the human Hsp90 complex.

## 8. PfAha1 (Activator of Hsp90 ATPase/PF3D7_0306200)

The Aha1 co-chaperone binds to the MD and stimulates Hsp90 ATPase activity, promoting client protein activation (Figure 1) [105]. PfAha1 was found using split ubiquitin assays [63] (Table 1). Employing GST pull-down assays, PfAha1 binds PfHsp90 in a manner dependent on MgCl_2_ and ATP [63]. PfAha1 competes with Pfp23 to interact with PfHsp90 under similar conditions [57]. In contrast to the Pfp23–PfHsp90 interaction, where Pfp23 has an inhibitory effect on the ATPase activity of PfHsp90, PfAha1 stimulates the ATPase activity of PfHsp90 [63], consistent with the function of the human homolog [105]. It was observed by computational modelling that residue N108 in PfAha1 is critical for interaction with PfHsp90, and the mutation of N108 to alanine leads to reduced stimulation of the ATPase activity of PfHsp90 [63]. The PfAha1–PfHsp90 interaction is likely polar in nature, as it is disrupted by high salt concentration. PfAha1 most likely plays a role in the maturation of PfHsp90 client proteins [57]. Furthermore, the presence of PfAha1 suggests that, despite the higher basal ATPase activity of PfHsp90 compared to hHsp90, client release from late stage chaperone complexes is still regulated by ATPase stimulation.

## 9. PfPP5 (Protein Phosphatase 5/PF3D7_1355500)

PP5 is a TPR-containing co-chaperone that regulates the Hsp90 chaperone cycle through the dephosphorylation of Hsp90 or co-chaperones, such as Cdc37 [106]. Degenerate deoxyoligonucleotide primers were used to identify the protein phosphatase protein in *P. falciparum* for the first time [107] (Table 1). Sequence analysis reveals that PfPP5 has a N-terminal TPR domain followed by a Ser/Thr phosphatase sequence at the C-terminal domain. The PfPP5 Ser/Thr domain is essential for phosphatase activity, and the TPR domain of the protein can act as a negative regulator of phosphatase activity. The N-terminal PfPP5 TPR domain is a potential anti-malaria target for the design of selective inhibitors [107]. This is because PfPP5 possesses an unusually long TPR domain with four TPR motifs, as opposed to the three usually observed in homologs of other species, including human. Using a PfPP5 antibody, both PfPP5 and PfHsp90 were co-immunoprecipitated, which implies that PfPP5 may be part of the Hsp90 chaperone complexes, as observed in mammals [64,65]. PP5 and Aha1 are important in many cellular processes in neurodegenerative diseases in association with Hsp90; therefore, it is important to study this co-chaperone in *P. falciparum* to understand its precise mechanism [108].

In yeast, Ppt1 (PP5 homologue) is demonstrated to specifically dephosphorylate Hsp82 [109]. The deletion of Ppt1 in yeast leads to the hyperphosphorylation of Hsp90 and the reduced efficiency of the Hsp90 chaperone system in activating client proteins (e.g., glucocorticoid receptors, v-Src, and Ste11). In addition, PP5/Ppt1 was also found to dephosphorylate another co-chaperone Cdc37 at the phosphorylated S13 residue, and modulate its activity in recruiting protein kinase clients to Hsp90 [106]. Hence, PP5/Ppt1 was proposed as a positive modulator for the activation of Hsp90 client proteins. In the case of *P. falciparum*, although PfPP5 interacts with PfHsp90 [107], it remains unclear whether PfPP5 exerts its phosphatase activity on PfHsp90. However, the presence of the PfPP5 phosphatase implies that the PfHsp90 complex undergoes phosphorylation by *P. falciparum* kinases.

## 10. PfCBP (Calcyclin-Binding Protein/PF3D7_0933200) and PfCns1 (Cyclophilin Seven Suppressor 1/PF3D7_1108900)

The calcyclin-binding protein (CBP), suppressor of G2 allele of Skp1 (Sgt1), cyclophilin seven suppressor 1 (Cns1), and tetratricopeptide repeat domain 4 (TTC4) all share significant sequence similarity, contain TPR domains, and are co-chaperones of Hsp90 [110,111,112]. While related, these co-chaperones each bind differently to Hsp90, and target selective sets of client proteins [57,60,66]. For example, Sgt1 associates with the N-terminus of Hsp90, and specifically recruits leucine-rich-repeat proteins [112]. Bioinformatics analyses applying protein domain homology, identified several putative PfHsp90 co-chaperones related to Sgt1/CBP and TTC4/Cns1, namely, PfCBP and PfCns1, respectively [94] (Table 1). However, further investigation is needed to confirm if these co-chaperones directly interact with PfHsp90 and modulate its chaperone function.

## 11. Cdc37 (Cell Division Cycle 37) Homolog Potentially Missing in *P. falciparum*

Cdc37 is involved in the recruitment of nascent or unstable kinases to Hsp90 for folding into their active conformation [113,114], and is known to be important for the activation of a diverse group of protein kinases (e.g., Cdk1, Cdk4, Akt, v-Src, Raf, and CK2) [115,116]. Indeed, as many as 65% of the kinases in yeast are reported to require Cdc37 for activation and stabilization [117]. In human cells, 60% of kinases interact with Hsp90, and the recognition of these kinases is mediated by Cdc37 [118]. As many of the kinases have essential signal transduction roles that regulate growth and development, Cdc37 is, thus, recognized as an important component of the Hsp90 chaperone machinery. In addition, Hsp90 chaperone activity itself is integrated with cellular proliferation by phosphorylation. It is, therefore, noteworthy that a Cdc37 homolog has not been found in *P. falciparum* (Table 1). This could mean that other *P. falciparum* co-chaperones are able to functionally compensate for the lack of Cdc37, especially since critical kinases known to associate with Cdc37, such as Cdk1 (PfPK5; MAL13P1·279), Akt (PfPKB; PFL2250c), and CK2 (PfCK2; PF11_0096), are found in *P. falciparum* [94]. The Cdc37 ortholog may be divergent from that of humans and, hence, has not been identified based on sequence identity. Alternatively, *P. falciparum* kinases may have differing chaperone requirements, meaning they can enter the cycle in the absence of Cdc37, or are less reliant on Hsp90 for function.

## 12. Conclusions

This review suggests that the Hsp90 chaperone, and its associated co-chaperone complexes in *P. falciparum,* are broadly conserved in comparison to other organisms. PfHsp90 displays biochemical differences to hHsp90, which may be targeted for selective inhibition. Importantly, Hsp90 does not function alone, and appropriate proteostasis requires that the chaperone be fine-tuned by co-chaperones. The core co-chaperones regulating client entry, ATPase activity, and Hsp90 conformational regulation at the early, intermediate, and late stages of the chaperone cycle are broadly conserved in *P. falciparum*. However, there are two notable differences that may indicate important areas for future study and evaluation of therapeutic potential.

The first is the presence of two p23 orthologs in *P. falciparum*. While both of these isoforms function similar to p23 in the Hsp90 complex, there are differences in client protein specificity and ATPase inhibition. The requirement of both isoforms for parasite viability, and their individual importance in the PfHsp90 chaperone cycle, have not yet been determined. Since one of the functions of p23 is to inhibit Hsp90 ATPase activity, it may be speculated that the two isoforms arose because of the higher basal ATPase activity of PfHsp90. Given that Pfp23A inhibits the PfHsp90 ATPase activity more than Pfp23B, and that the folding and activation of different clients may require different cycle timing, the two p23 isoforms may have evolved to assist different client protein groups (i.e., the higher ATPase activity of PfHsp90 may allow for more inhibitory steps in the chaperone cycle). A detailed analysis of the co-chaperone functions of these p23 isoforms in vitro and in the parasite would be useful in determining if mechanistic differences do exist, and if they have therapeutic potential.

The second notable difference is the apparent absence of a Cdc37 ortholog in *P. falciparum*. However, since Cdc37 orthologs were identified in other obligate intracellular protozoan parasites (e.g., *Theileria annulata* and *Cryptosporidium parvum*) [119], deeper scrutiny of the *P. falciparum* genome is required. Cdc37 is regarded as one of the most important therapeutic Hsp90 co-chaperones, because of its role in regulating kinase entry into Hsp90 complexes. Kinases are considered important therapeutic targets in both cancer (focusing on human kinases) and malaria, and kinase inhibitors form one of the largest classes of approved drugs. The *P. falciparum* kinome was recently updated, confirming that its kinome is considerably smaller (98 members compared to 497 members in the human kinome) and divergent (38% unique; 46% potentially unique; and 16% human homologs) from that of humans [120]. Therefore, the apparent lack of a Cdc37 ortholog, or the presence of a yet to be identified divergent Cdc37 ortholog or functional equivalent, is likely to reflect differences in the folding requirements of the *P. falciparum* kinome by the Cdc37–PfHsp90 co-chaperone–chaperone machinery. Furthermore, the co-evolution of PfHsp90 and the kinome could have resulted in reduced dependency on a canonical Cdc37 for kinase activation. Indeed, there is evidence that Hsp90 may be able to activate kinases in the absence of Cdc37 [121,122]. Importantly, no study has demonstrated that *P. falciparum* kinases require PfHsp90 in a mechanism analogous to their yeast and human orthologs. Given the importance of kinases to drug discovery, and the fact that many *P. falciparum* kinases are being evaluated as drug targets, it would be interesting to identify a bona fide PfHsp90 kinase client. This could easily be done using available Hsp90 inhibitors in malaria parasite cell lines expressing GFP-tagged kinases. Validation of at least one PfHsp90 kinase client would subsequently support efforts to determine whether or not Cdc37 exists in the malaria parasite. This would be interesting not only from a fundamental perspective, but also in terms of identifying a selective therapeutic target for simultaneous inhibition of multiple kinases.

Taken together, both the conservation and differences in the co-chaperone complexes of PfHsp90 suggest that, as in non-communicable diseases [34], targeting Hsp90–co-chaperone interactions is an exciting new area of research that can both extend our understanding of proteostasis, and identify novel approaches for inhibition.

## Figures and Tables

**Table 1:** Co-chaperones of Hsp90 in *Homo sapiens* and *Plasmodium falciparum*.

Humans	*P. falciparum*	Known Functions	References
Hop	PfHop (PF3D7_1434300)	Early stage co-chaperone; binds Hsp90 at C-terminus; adaptor for Hsp70 and Hsp90; inhibits ATPase activity	[54,55]
Tah1	PfRPAP3/PfTah1 (PF3D7_0213500)	Component of Rvb1-Rvb2-Tah1-Pih1 (R2TP) complex	[56]
Pih1	PfPih1 (PF3D7_1235000)	Component of Rvb1-Rvb2-Tah1-Pih1 (R2TP) complex	[56]
Cyp40	PfCyp40 (PF3D7_1111800	Peptidyl prolyl-cis/trans-isomerase	[57]
FKBP38	PfFKBP35 (PF3D7_1247400)	Peptidylprolyl-cis/trans-isomerase	[58,59]
TTC4	PfCns1 (PF3D7_1108900)	TTC4 is known for its interaction with cyclophilin; activated ATPase activity of Hsp70 by binding at TPR domain	[57,60]
p23	Pfp23A (PF3D7_1453700) Pfp23B (PF3D7_0927000)	Late stage co-chaperone, stabilizes closed Hsp90 confirmation; inhibits ATPase activity of Hsp90	[61,62]
Aha1	PfAha1 (PF3D7_0306200)	Potent ATPase activator of Hsp90; promotes client maturation	[57,63]
PP5	PfPP5 (PF3D7_1355500)	Phosphatase activity	[64,65]
Sgt1	PfCBP (PF3D7_0933200)	Kinetochore assembly	[66]
Cdc37	Not found	Early stage co-chaperone; kinase-specific co-chaperone and inhibits ATPase activity of Hsp90	[67]
